# From Traditional Usage to Pharmacological Evidence: A Systematic Mini-Review of Spina Gleditsiae

**DOI:** 10.1155/2016/3898957

**Published:** 2016-06-28

**Authors:** Jiayu Gao, Xiao Yang, Weiping Yin

**Affiliations:** ^1^School of Chemical Engineering and Pharmaceutics, Henan University of Science & Technology, Luoyang, Henan 471023, China; ^2^No. 1 Hospital, Henan University of Science & Technology, Luoyang, Henan 471000, China

## Abstract

Spina Gleditsiae is an important herb with various medicinal properties in traditional and folk medicinal systems of East Asian countries. In China through the centuries, it has been traditionally used as a source of drugs for anticancer, detoxication, detumescence, apocenosis, and antiparasites effects. Recently, an increasing number of studies have been reported regarding its chemical constituents and pharmacological activities. To further evidence the traditional use, phytochemicals, and pharmacological mechanisms of this herb, a systematic literature review was performed herein for Spina Gleditsiae. The review approach consisted of searching several web-based scientific databases including PubMed, Web of Science, and Elsevier using the keywords “Spina Gleditsiae”, “Zao Jiao Ci”, and “*Gleditsia sinensis*”. Based on the proposed criteria, 17 articles were evaluated in detail. According to the reviewed data, it is quite evident that Spina Gleditsiae contains a number of bioactive phytochemical components, which account for variety medicinal values including anticancer, anti-inflammatory, antiatherogenic, antimicrobial, antiallergic, and antivirus activities. The phytochemical and pharmacological studies reviewed herein strongly underpin a fundamental understanding of herbal Spina Gleditsiae and support its ongoing clinical uses in China. The further phytochemical evaluation, safety verification, and clinical trials are expected to progress Spina Gleditsiae-based development to finally transform the traditional TCM herb Spina Gleditsiae to the valuable authorized drug.

## 1. Introduction

Spina Gleditsiae, also known as Zao Jiao Ci (in Chinese), is the dry thorn of* Gleditsia sinensis* Lam (*G. sinensis*).* G. sinensis* is a perennial shrub native to China and widely grows in the basin of Yellow and Yangtze River, as well as Guangdong, Guangxi, Guizhou, and Yunnan provinces of China. Its different parts, named Da Zao Jiao (fruit), Zhu Ya Zao (anomalous fruit), Zao Jia Zi (seed), Zao Jia Ye (leave), Zao Jiao Ci (thorn), and Zao Jiao Gen Pi (radix cortexes), have long been used in traditional Chinese medicine (TCM). Among them, Gleditsiae Sinensis Fructus (Da Zao Jiao), Gleditsiae Fructus Abnormalis (Zhu Ya Zao), and Spina Gleditsiae (Zao Jiao Ci) are officially recorded in the Chinese Pharmacopoeia [1]. The herbal name of Spina Gleditsiae was first documented in* Tu Jing Ben Cao* by Su in about 1061 AD [2]. It has been an important herbal medicine with various medicinal properties in traditional and folk medicinal systems of East Asian countries, such as China, Korea, and Japan. In China through the centuries, it has been traditionally used as a source of drugs for the detoxication, detumescence, apocenosis, and antiparasites effects [1]. Moreover, Spina Gleditsiae is also claimed to be used as the main active ingredient in many TCM anticancer formulae used in clinics of China [3].

Previously, the knowledge of either phytochemistry or pharmacology of Spina Gleditsiae underlying its traditionally medicinal uses is rarely summarized and systematically analyzed. In spite of that, there are an increasing number of studies that have been reported recently regarding the active chemical constituents and pharmacological activities of Spina Gleditsiae. To support the further phytopharmacological research, drug development, and clinical use of this herb in laboratories, pharmaceutical industries, or TCM hospitals, this present work herein aims to perform a systematic literature review on the traditional use, phytochemistry, and pharmacological aspects of Spina Gleditsiae. The review searched a number of electronic databases, including PubMed, Web of Science, and Elsevier, up to the date of 13 May 2016. The keywords included Spina Gleditsiae, Zao Jiao Ci, and* Gleditsia sinensis*. Searching was limited to articles only in English. The irrelevant papers, reviews, patents, abstracts, case reports, and abstracts in symposium and congress were excluded. The articles were reviewed by two authors independently to determine compatibility with the inclusion criteria above, and 17 articles were eligible to be evaluated in this paper.

## 2. Traditional Usage of Spina Gleditsiae

The high medicinal importance of Spina Gleditsiae is widely known in traditional and folk medicinal systems in East Asian countries such as China, Korea, and Japan. In herbal medicine, Spina Gleditsiae extracts or its isolated constituents have been known to demonstrate antimutagenic [4], antimicrobial [5], anti-HIV [6], anti-inflammatory [7], antitumour [8], and cardioprotective [9] activities.

In China, Spina Gleditsiae is traditionally used by the TCM doctors as a source of drugs for treating symptoms associated with acute mastitis, skin ulcer, inflammation of the sublingual soft tissue, acute tonsillitis, and cancer [10]. According to the* Compendium of Materia Medica*, one of the most authoritative TCM encyclopedias, Spina Gleditsiae is able to treat scrofula, dysuria, acute mastitis, and retained afterbirth [11]. In the official* Pharmacopoeia of the People's Republic of China*, the functions of Spina Gleditsiae include expelling phlegm, detoxication, apocenosis, and expelling parasite, and it is suggested for use in the treatment of ulcers, sepsis, scabies, and Hansen's disease [1]. For the inflammation-related treatment, the patients are typically instructed by TCM doctors to swallow the 15–25 g powder of Spina Gleditsiae twice per day until remission. The cancer patients are specially instructed to prepare the decoction at home by boiling Spina Gleditsiae-based herbal mixtures in water (once or twice to produce 300–400 mL). The decoctions are then self-administered 2-3 times daily. According to the report in literature, Spina Gleditsiae is also widely distributed in the Gyeongju city area in Korea. It is used in Korea for treatment of carbuncle, swelling, suppuration, scabies, and skin diseases [12]. Clearly, the bioactive secondary metabolites of the thorn of* G. sinensis* are the basic functional units to possess those pharmacological effects of the herbal Spina Gleditsiae.

## 3. Phytochemical Constituents of Spina Gleditsiae

**Figure 1:**
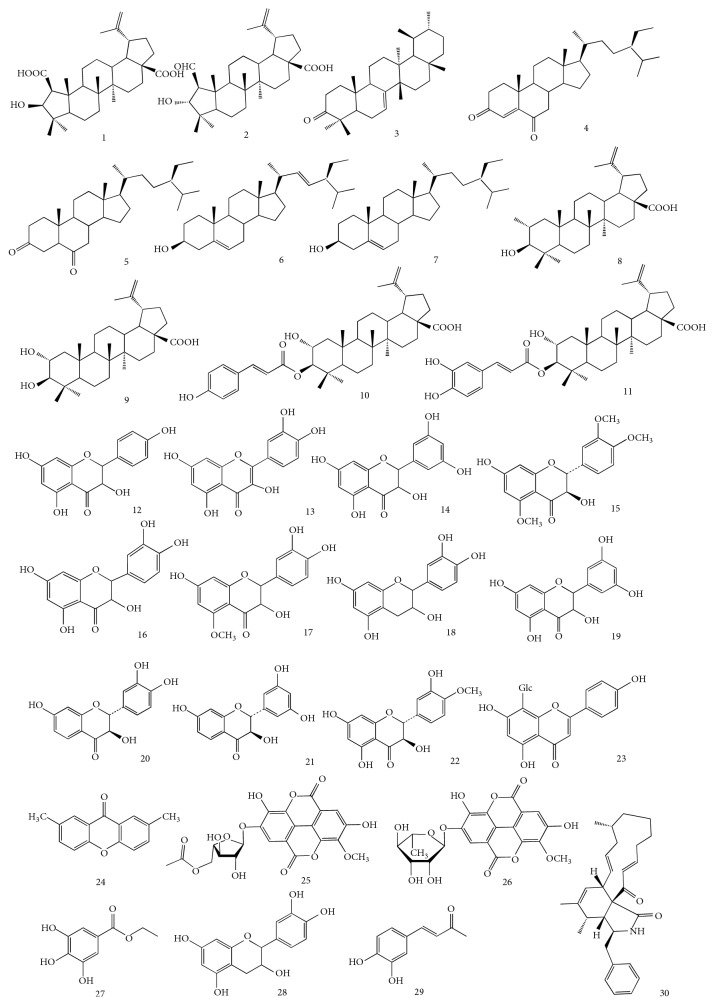
Compounds isolated from Spina Gleditsiae.

Spina Gleditsiae is rich in secondary metabolites, which are discovered to be responsible for exhibiting its medicinal activities. So far, there are totally 30 compounds belonging to five major groups of secondary metabolites, including triterpene, sterol, flavonoid, phenolic, and alkaloid, which have been isolated and elucidated from Spina Gleditsiae. The chemical structures, previously reported from this herbal medicine, are shown in Figure 1.

### 3.1. Triterpenes and Sterols

*G. sinensis*, particularly its fruit part, is rich in triterpenes [12]. However, there are also three triterpenes to be isolated from the thorn of* G. sinensis*. They were found to be 2*β*-carboxyl,3*β*-hydroxyl-norlupA(1)-20(29)-en-28-oic acid (1), zizyberanalic acid (2) [6], and D:C-friedours-7-en-3-one (3) [4], respectively.

Total eight sterols, including four lanostane-type and four lupane-type, were identified from Spina Gleditsiae. The lanostane-type compounds stigmast-4-ene-3,6-dione (4), stigmast-3,6-dione (5), stigmasterol (6), and *β*-sitosterol (7) were reported by Lim and his colleagues in 2005 [4]. Two years later, Li et al. found four lupine-type sterols, betulic acid (8), alphitolic acid (9), 3-O-trans-p-coumaroyl alphitolic acid (10), and 2-hydroxypyracrenic acid (11) [6].

### 3.2. Flavonoids

Flavonoids, the class of chemicals with multiple medicinal properties, are surprisedly not found abundantly from Spina Gleditsiae in previous studies of literature. Only three common known flavones, dihydrokaempferol (12), quercetin (13), and 3,3′,5′,5,7-pentahydroflavanone (14), were reported by Zhou's group [5]. However, our groups expanded this knowledge recently and isolated one new flavonoid (2R,3R)-5,3′,4′-trimethoxyl-7-hydroxyl-flavanonol (15), together with another nine flavonoids 5,7,3′,4′-tetrahydroxyl-flavanonol (16), 5-methoxyl-3′,4′,7-trihydroxylflavanonol (17), epicatechin (18), 5,7,3′,5′-tetrahydroxyl-flavanonol (19), fustin (20), (2R,3R)-7,3′,5′-trihydroxylflavanonol (21), (2R,3R)-5,7,3′-trihydroxyl-4′-methoxyl-flavanonol (22), 5,7,4′-trihydroxylflavone-8-C-glucopyranose (23), and 2,7-dimethyl-xanthone (24) from Spina Gleditsiae for the first time [13].

### 3.3. Phenolics and Alkaloids

Two ellagic acid glycosides, 3-O-methylellagic acid-4′-(5′′-acetyl)-*α*-L-arabinofuranoside (25) and 3-O-methylellagic acid-4′-O-*α*-L-rhamnopyranoside (26), and three phenolic acid relatives, ethyl gallate (27), (−)-epicatechin (28), and caffeic acid (29), were reported so far to be isolated from the spines of* G. sinensis* [5, 14]. In addition, one alkaloid, cytochalasin H (30), was obtained by Lee et al. through activity-guided fractionation on Spina Gleditsiae [15].

## 4. Pharmacological Activities

Spina Gleditsiae has been well studied for its multiple biological activities contributed by the presence of the wide array of bioactive phytochemicals summarized above. The bioactive studies revealed that the extracts or active compounds of Spina Gleditsiae exhibited a wide spectrum of pharmacological activities such as anticancer, anti-inflammatory, antiatherogenic, antimicrobial, antiallergic, and antivirus activities.

### 4.1. Anticancer Activities

Lee and his colleagues firstly reported the anticancer effects of Spina Gleditsiae in 2009 [8]. In their study, the herbal ethanol extract significantly arrested the cell cycle at G2/M phase and inhibited the growth of human colon cancer HCT116 cells* in vitro* with IC_50_ at 600 *µ*g/mL. Moreover, the ethanol extract of Spina Gleditsiae also significantly reduced tumour size in HCT116 cell-xenografted tumour tissues. Both of these* in vitro* and* in vivo* activities were associated with the regulation of expression of ERK phosphorylation, p27, and matrix metalloproteinase-9 (MMP-9) [8]. The water extract of Spina Gleditsiae presented a similar inhibitory effect on HCT16 cell* in vitro* and xenografts* in vivo* models as reported by Lee et al. in 2010. The underlying mechanisms of this inhibition were correlated with increased p53 levels, downregulation of cyclins and cyclin-dependent kinases, and phosphorylation of ERK, p38 MAP kinase, and JNK [16]. Moreover, Lee's research group further reported that the ethanol extract of Spina Gleditsiae led to the growth inhibition with G1 phase cell cycle arrest at a concentration of 400 *µ*g/mL on human SNU-5 gastric cancer cells. The mechanisms involved the activation of p38 MAP kinase and subsequently the induction of p21WAF1 and the downregulation of cyclin D1/CDK4 and cyclin E/CDK2 complexes [17]. Furthermore, oral administration of the water extract of Spina Gleditsiae (25 mg/kg/day) was found to significantly reduce the size of a PC3 prostate cancer cell-xenografted tumour in another experiment [18]. Except the extracts, three individual compounds identified from Spina Gleditsiae have been reported to present cytotoxic effects to human SK-Hep-1 liver cancer cells* in vitro*. Among them, p-hydroxyl cinnamic aldehyde exhibited cytotoxicity with IC_50_ at a value as low as 38.18 *µ*M, while trans-coniferyl aldehyde and sinapaldehyde showed moderate effects with IC_50_ values of 58.11 and 54.61 *µ*M, respectively [19].

Angiogenesis is the process of new vessel formation from preexisting blood vasculature and is the key for continuous tumour growth. The antiangiogenesis activity of Spina Gleditsiae was firstly reported by Yi et al. in 2012. According to their report, the ethanol extract of Spina Gleditsiae was able to inhibit the proliferation of human umbilical vein endothelial cells (HUVEC)* in vitro*. Cell migration and tube formation were also significantly inhibited in a dose-dependent manner. New vessel formation of nude mice was reduced by the treatment of the ethanol extract of Spina Gleditsiae. The downregulation of proangiogenic proteins, endothelin 1 and matrix metallopeptidase 2, was determined to account for those* in vitro* and* in vivo* antiangiogenesis effects [20]. Furthermore, an antiangiogenesis compound, cytochalasin H, was identified from Spina Gleditsiae by Lee and his colleagues after two years. The growth and mobility of HUVEC were suppressed by cytochalasin H through decreasing expression of proangiogenic factor EDN1. Moreover, the compound could inhibit the proangiogenic protein induced formation of new blood vessels and the tumour growth* in vivo*. Taken together, these results revealed that Spina Gleditsiae and its compound cytochalasin H were potential antiangiogenic cancer candidates [15, 21].

### 4.2. Anti-Inflammatory Activities

As Spina Gleditsiae is traditionally used for the treatment of inflammatory diseases, its anti-inflammatory activities have been studied to support this clinical use. Ha et al. reported that the aqueous extract of Spina Gleditsiae could effectively inhibit the production of lipopolysaccharide- (LPS-) induced nitric oxide (NO) and the expression of inducible NO synthase (NOS) in RAW 264.7 macrophages. The mechanisms underlying this effect included the suppression of NF-кB activation, phosphorylation and degradation of IкB-*α*, and phosphorylation of extracellular signal-regulated kinase 1/2 (ERK 1/2) and c-Jun N-terminal kinase (JNK) [7]. Moreover, the ethyl acetate fractions of Spina Gleditsiae were also found to reduce the production of NO and prostaglandin E2 in RAW 264.7 and the production of thymus- and activation-regulated chemokine in HaCaT cells* in vitro*. Six compounds including (+)-catechin, (−)-epicatechin, eriodictyol, quercetin, caffeic acid, and ethyl gallate at least partially account for this effect [22].

### 4.3. Antiatherogenic Activities

The ethanol extract of Spina Gleditsiae exerted an inhibitory effect on the proliferation and TNF-*α*-induced MMP-9 expression of vascular smooth muscle cells (VSMC)* in vitro*, which could explain the therapeutic use of this herb for the treatment of atherosclerosis disease in China. The ERK 1/2, p38 MARK, and JNK activation, together with the suppression of G2/M cell cycle regulators cyclin B1, Cdc2, and Cdc25c, were identified to be responsible for the antiatherogenic activity of Spina Gleditsiae [9]. Furthermore, Park and his colleagues reported that the ethanol extract of Spina Gleditsiae could induce p27KIP1-mediated G1 cell cycle arrest and reduced Akt phosphorylation and MMP-9 expression by suppressing the binding activities of NF-кB, AP-1, and Sp-1, thus leading to growth inhibition of VSMC and the suppression of migration and invasion [23].

### 4.4. Antimicrobial Activities

In two early studies, the antibacterial and antifungal activities of Spina Gleditsiae were identified by Zhou's group in 2007. Compared with the diameter of inhibitory zones of streptomycin against* Bacillus subtilis* (21.5 mm) and* Xanthomonas vesicatoria* (13.8 mm), the herbal ethanol extract showed moderate effects with diameters of 9.5 and 9.2 mm, respectively. Moreover, the isolated compounds quercetin, 3,3′,5′,5,7-pentahydroflavanone, and caffeic acid demonstrated the antibacterial effects against* Bacillus subtilis* with minimal inhibitory concentration (MIC) of 0.5, 0.5, and 0.125 mg/mL and against* Xanthomonas vesicatoria* with MIC of 0.75, 0.75, and 0.125 mg/mL, respectively. Ethyl gallate and dihydrokaempferol also inhibited* Bacillus subtilis* with MIC at 1.00 mg/mL [5]. Zhou and his colleagues further reported the other two compounds 3-O-methylellagic acid-4′-(5′′-acetyl)-*α*-L-arabinofuranoside and 3-O-methylellagic acid-4′-O-*α*-L-rhamnopyranoside from Spina Gleditsiae showed significant antifungal activity against the spore germination of rice blast fungus* Magnaporthe grisea* with IC_50_ at 13.56 and 16.14 *µ*g/mL, respectively [14].

### 4.5. Antiallergic Activities

The water extract of Spina Gleditsiae was reported to inhibit both of the systemic anaphylaxis induced by compound 48/80 and the local anaphylaxis activated by anti-DNP IgE in rats. The herbal extract was found to suppress the histamine release from peritoneal mast cells (RPMC). The level of cyclic AMP of RPMC could transiently increase fourfold compared with that of basal cell under Spina Gleditsiae treatment [24]. Moreover, the ethanol extract of Spina Gleditsiae could inhibit the accumulation of eosinophils in airways and reduce the levels of IL-4 and IL-5 in bronchoalveolar lavage fluid (BALF) and IgE in BALF and plasma of* in vivo* murine model. The mechanism of this effect was partially mediated by the reduction of oxidative stress and airway inflammation [25].

### 4.6. Antivirus Activities

Li et al. evaluated 2*β*-carboxyl,3*β*-hydroxyl-norlupA(1)-20(29)-en-28-oic acid, the compound isolated from Spina Gleditsiae, against HIV-1 replication in C8166 cells and found that the compound processed the potent anti-HIV activity with EC_50_ of less than 0.064 *µ*g/mL [6].

## 5. Clinical Usage in China

Though there were no clinical reports and trials published in English literature, as a matter of fact, Spina Gleditsiae is currently used as a clinical drug in many TCM hospitals in China. Used either as a single medicine or as an ingredient of the TCM formula, Spina Gleditsiae has been reported to treat facial paralysis [26], chronic pharyngitis, chronic synovitis, hyperplasia of mammary glands, hyperlipidemia [27], urinary retention [28], acne [29], erysipelas [30], prostatitis [31], ischialgia [32], and cancer [33] in clinics of China. The therapeutic outcomes of those usages were mostly claimed as being significantly effective in the Chinese clinical reports based on the single or small number of clinical cases. The side effects and safety evaluation of those usages unfortunately remained as an ongoing challenge in those reports. However, as a significant traditional Chinese medicine, the comprehensive well-controlled and double-blind clinical trials are urgently needed to determine the efficacy, safety, and side effects of clinical usage of Spina Gleditsiae. The scientifically evidence-based clinical reports, especially in English, will attract more interests internationally and thus expand the research and usage of this medicine.

## 6. Conclusion and Future Prospect

Spina Gleditsiae has been used as traditional herbal medicine for centuries in China, and an increasing number of studies have been reported regarding its chemical constituents and pharmacological activities recently. According to the data accumulated in this review, it is quite evident that Spina Gleditsiae contains a number of bioactive phytochemical components, which account for variety medicinal values. The development of effective new drugs from Spina Gleditsiae thus can be considered in the treatment of diseases related to its main pharmacological activities. Meanwhile, although photochemical and pharmacological studies on Spina Gleditsiae have received great interest, most of the current studies only focused on the crude extracts, and there is not enough evidence regarding purified compounds and their pharmacological actions. Therefore, further studies are highly suggested to elucidate the bioactive mechanisms of the exact pure constituents. Also, various pharmacological activities of Spina Gleditsiae or its compounds were conducted to test either* in vitro* or* in vivo* assays, and no clinical trials have been reported in English so far. Therefore, the clinical efficacy, side effects, and safety of this medicine remain unknown. Comprehensive well-controlled and double-blind clinical trials are therefore urgently needed.

Overall, the phytochemical and pharmacological studies reviewed herein strongly underpin a fundamental understanding of herbal Spina Gleditsiae and support its ongoing clinical uses in China. The further phytochemical evaluation, safety verification, and clinical trials are expected to progress Spina Gleditsiae-based development to finally transform the traditional TCM herb Spina Gleditsiae to the valuable authorized drug.
